# Urachal Cyst, a Rare Cyst with Multiple Complications

**DOI:** 10.1100/tsw.2008.45

**Published:** 2008-02-25

**Authors:** Amr Hawary, Peter Duffy

**Affiliations:** Lancaster Royal Infirmary, UK

**Keywords:** urachal, anomalies, cyst, adenocarcinoma

## Abstract

Urachal cysts are rare, can be one of the forgotten causes of abdominal pain, and can present as adenocarcinoma of the bladder, along with many other presentations. There is a real need for physicians, general surgeons, and urologists to be acquainted with the different presentations and management of this rare condition.

